# Supplementary material for: Artificial Intelligence in Detection, Management, and Prognosis of Bone Metastasis: A Systematic Review
Source: Cancers (Basel). 2024 Jul 29;16(15):2700. doi: 10.3390/cancers16152700 (PMC11311270; doi:10.3390/cancers16152700)
Supplement: Supplementary file 1 [file cancers-16-02700-s001.zip › cancers-3089253-supplementary.pdf]

LIST OF ACRONYMS

- AI: artificial intelligence
- CNN: convolutional neural networks
- DL: deep learning
- ML: machine learning
- ANN: artificial neural network
- DNN: deep neural network
- CT: computed tomography
- MRI: magnetic resonance imaging
- BS: bone scintigraphy
- PET-CT: positron emission tomography and computed tomography
- SPECT: single photon emission computed tomography
- SERS: surface-enhanced Raman spectroscopy
- PSMA: prostate-specific membrane antigen
- PSA: prostate-specific antigen
- Tc-MDP: Technetium-99m methylene diphosphonate
- CAD: computer-assisted diagnostic
- GBM: Gradient boosting machine
- XGB: extreme gradient boosting
- DT: Decision tree
- PC: parallelepiped classification
- SVM: support vector machine
- CBN: Causal Bayesian networks
- BSI: Bone Scan Index
- KNN: k-nearest neighbor
- IFV: Irregular flux viewer
- RPN: Region proposal network
- MLP: Multilayer Perceptron
- AUC: area under the curve
- ROC: receiver operating characteristic
- SUV: standardized uptake value
- MTV: metabolic tumor volume
- TLG: total lesion glycolysis
- APV: alkaline phosphatase velocity
- NBC: Naive Bayes classifiers

- SORG: stochastic gradient boosting
- DSC: dice similarity coefficient
- GLCM: Gray level Co-occurrence Matrix
- DWI: diffusion-weighted imaging
- DWIBS: diffusion-weighted whole-body imaging with background body signal suppression
- RF: random forest
- LR: logistic regression
- SRE: skeletal related events
- BTA: bone-targeting agents
- CCS: cancer-specific survival
- OS: overall survival
- DFS: disease-free survival
- MST: median survival time
- SCLC: small cell lung cancer
- GEO: Gene Expression Omnibus
- LF: label-free
- SERS: surface-enhanced Raman scattering
- BAP: bone-specific-alkaline phosphatase
