# Supplementary figures and images for: Artificial Intelligence in Detection, Management, and Prognosis of Bone Metastasis: A Systematic Review
Source: Cancers (Basel). 2024 Jul 29;16(15):2700. doi: 10.3390/cancers16152700 (PMC11311270; doi:10.3390/cancers16152700)

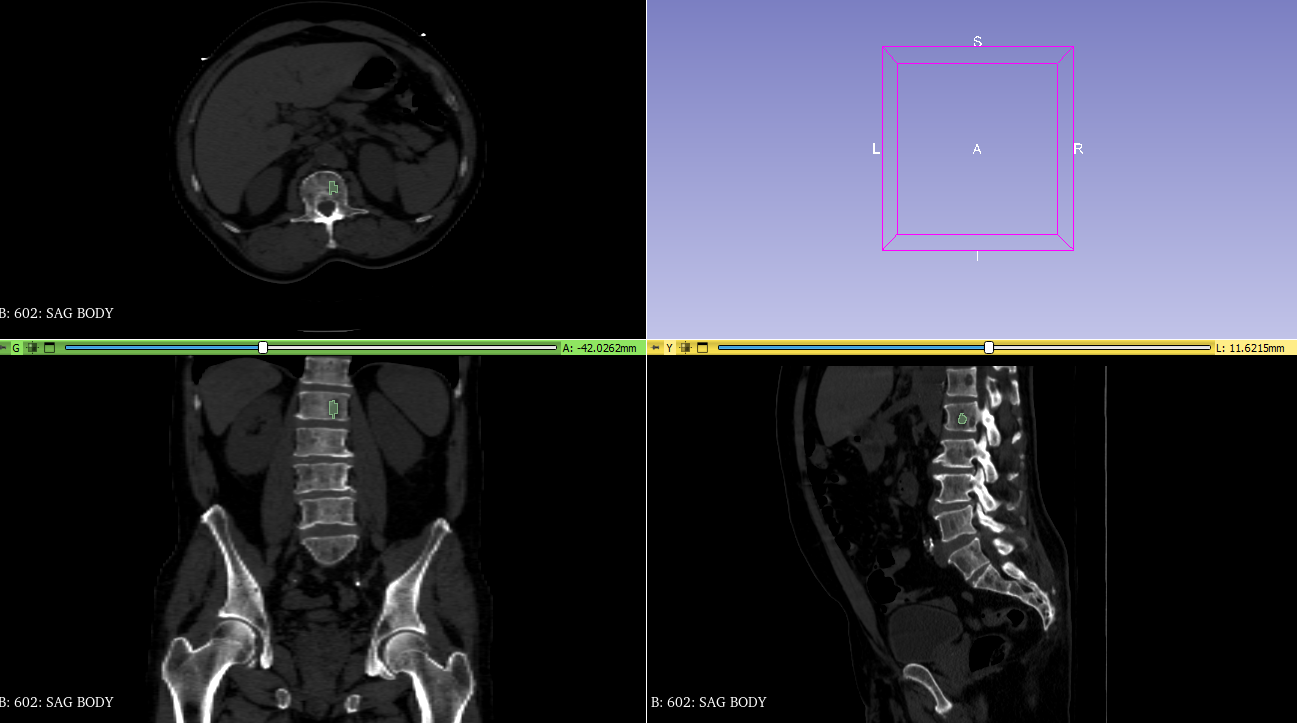

Supplement: Supplementary file 1 [file cancers-16-02700-s001.zip › cancers-3089253-figures.png]
